# Concurrent photoacoustic and ultrasound microscopy with a coaxial dual-element ultrasonic transducer

**DOI:** 10.1186/s42492-018-0003-4

**Published:** 2018-09-05

**Authors:** Yuqi Tang, Wei Liu, Yang Li, Qifa Zhou, Junjie Yao

**Affiliations:** 1 Department of Biomedical Engineering, Duke University, Durham, NC 27708 USA; 2 Department of Ophthalmology, Department of Biomedical Engineering, University of Southern California, Los Angeles, CA 90089 USA

**Keywords:** Photoacoustic microscopy, Ultrasound microscopy, Concurrent imaging, Coaxial dual-element ultrasonic transducer

## Abstract

Simultaneous photoacoustic and ultrasound (PAUS) imaging has attracted increasing attention in biomedical research to probe the optical and mechanical properties of tissue. However, the resolution for majority of the existing PAUS systems is on the order of 1 mm as the majority are designed for clinical use with low-frequency US detection. Here we developed a concurrent PAUS microscopy that consists of optical-resolution photoacoustic microscopy (OR-PAM) and high-frequency US pulse-echo imaging. This dual-modality system utilizes a novel coaxial dual-element ultrasonic transducer (DE-UST) and provides anatomical and functional information with complementary contrast mechanisms, achieving a spatial resolution of 7 μm for PA imaging and 106 μm for US imaging. We performed phantom studies to validate the system’s performance. The vasculature of a mouse’s hind paw was imaged to demonstrate the potential of this hybrid system for biomedical applications.

## Background

Photoacoustic (PA) imaging uses optical absorption as the contrast mechanism and can thus visualize the optical properties of tissue [1]. One of the most widely imaged endogenous chromophores is hemoglobin in the red blood cells, which provides high contrast and high resolution PA images of vasculature in vivo [2–4]. However, the contrast mechanism of PA imaging limits that only selective biomolecules are visible, it is therefore useful to complement PA imaging with ultrasound (US) imaging, which can reveal tissue morphology at depths of up to tens of centimeters [5–7]. Since US imaging derives contrast from echogenicity and differing mechanical properties of tissue, it can provide general structural information that is typically absent from PA images [8–10]. Thus, concurrent PA and US imaging (PAUS) has gained increasing interest in the last decade for both preclinical and clinical applications [6, 8, 11–14].

As a dual-modality imaging system, the merits of PAUS imaging can be summarized as follows: 1) PA imaging provides functional and molecular information about tissue and US imaging enables anatomical localization [11, 13]. This allows the integrated PAUS system to identify structural and functional abnormalities and diseases [15], enhance the sensitivity and specificity of early stage cancer diagnosis and metastases detection [11, 13, 16], and guide interventional procedures such as needle injection and laser ablation with higher contrast [8, 13, 17]. 2) PA imaging is inherently compatible with US imaging, as both modalities acquire acoustic signals. With the commercial programmable US systems currently available, PA imaging can be readily integrated into an US system [12, 14, 18, 19], and PA and US images can be easily co-registered. 3) Moreover, morphologic information provided by US imaging such as tissue boundaries, speed of sound, and acoustic attenuation may aid in the reconstruction of PA images [18, 20–22].

However, the reported PAUS systems mostly rely on the commercially available ultrasound transducer probes for acoustic detection, which generally have frequencies below 10 MHz [8, 12–14]. The low frequency ultrasound leads to relatively deep penetration, at the expense of spatial resolution. For the other PAUS systems that use high-frequency ultrasound detection, the spatial resolution is much improved [23–25]. However, the detection of the PA and US signals is typically separated, resulting in a long imaging time.

In this work, we will present a truly concurrent photoacoustic and ultrasound (PAUS) microscopy system that provides automatically co-registered PA and US images using a novel coaxial dual-element ultrasonic transducer. This PAUS microscopy system can reveal detailed structural and functional information simultaneously, by acquiring the PA and US signals simultaneously at each lateral position. We have performed phantom and animal studies to demonstrate the hybrid imaging capability. For readers without access to the customized dual-element ultrasonic transducer, we have also provided an alternative engineering solution using two commercial focused ultrasonic transducers, at the expense of the system complexity and imaging depth.

## Methods

**Fig. 1:**
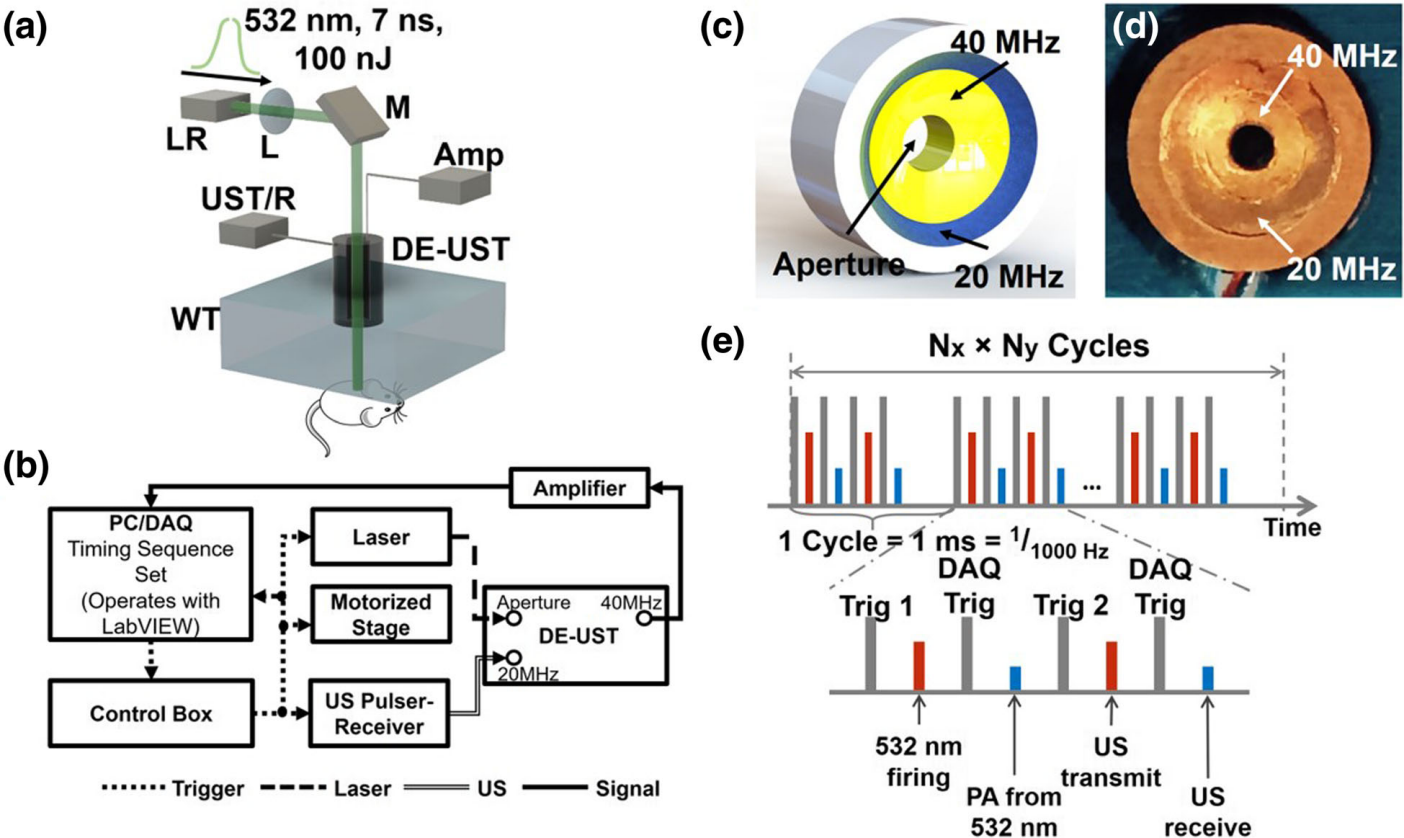
Concurrent PAUS microscopy. **a** The schematic of the PAUS microscopy system. LR, laser; L, lens; M, mirror; UST/R, ultrasonic pulser-receiver; Amp, amplifier; WT, water tank with transparent bottom. The mouse was placed under a small water tank on a 3D motorized translation stage (not shown) with the hind leg fixed on a sample holder. **b** Experimental timing sequence chart. A LabVIEW program sends out triggers to the laser, ultrasonic pulser-receiver, stage, and DAQ. **c**, **d** Structure and photo of the DE-UST with a central aperture. The high frequency element (40 MHz) is inside and the low frequency element (20 MHz) is outside. **e** Detailed timing sequence. Trigger 1 is for 532 nm laser firing, followed by a DAQ trigger that starts data acquisition for the PA signal. Trigger 2 is for US transmission, followed by a DAQ trigger that starts data acquisition for the US signal. The A-line acquisition frequency is 1000 Hz, and the sampling frequency for each A-line is 250 MHz

Figure 1a, b show the schematic of the concurrent PAUS microscopy system. An Nd: YAG fiber laser (VPFL-G-20, V-Gen, Tel Aviv, Israel) delivered a 7 ns pulse with a pulse energy of 100 nJ at a wavelength of 532 nm. The beam was first focused by a convex lens with a focal length of 50 mm (AC127–050-A, Thorlabs, Newton, NJ, USA), then passed through the aperture of a ring-shaped coaxial dual-element ultrasonic transducer (DE-UST), illuminating the target with a beam diameter of 7 μm at the focal point. The customized DE-UST has both a low frequency (20 MHz) and a high frequency element (40 MHz). The two transducer elements are concentrically and confocally arranged into a single device, as shown in Fig. 1c, d. Both transducer elements were lapped from 500 μm lithium niobate plates (Boston Piezo-Optics, Bellingham, MA, USA) to the half-wavelength thickness. The lapped material was electroplated with a chrome/gold (Cr/Au) layer on both sides. A conductive backing layer was casted using conductive silver epoxy. The materials were bound together with epoxy to form a single cylinder and pressed by a metal ball to form the same focal length of 11.25 mm. Both transducer elements have a − 6 dB bandwidth of 78%. A central aperture with a diameter of 2 mm was used to deliver light. The outer diameter of the high-frequency (40 MHz) transducer element is 7.9 mm and the outer diameter of the low-frequency (20 MHz) transducer element is 11.2 mm.

The 20 MHz transducer element was connected to an ultrasonic pulser-receiver (5800PR, Olympus, Waltham, MA, USA) for ultrasound transmission and the 40 MHz transducer element was connected to an amplifier for receiving both US and PA signals. The lateral resolution of PA imaging is determined by the optical focus of 7 μm. The 40 MHz transducer element provides an axial resolution of 36 μm for PA and a lateral resolution of 106 μm for US. The 20 MHz transducer element was used for US transmission for a deeper penetration. Although the 20 MHz transducer element can be used for both US transmission and detection, it is preferable to receive the PA and US signals using the 40 MHz transducer element for the following two reasons: (1) The 40 MHz element naturally attenuates the reflected 20 MHz US signals, which are typically more than 100 times stronger than the PA signal, allowing both PA and US signals to be acquired in the same dynamic range without saturation or suppression; and (2) Both PA and US signals can be amplified and sampled by a single-channel amplifier and a single-channel data acquisition card (DAQ), reducing the cost of the imaging system.

To achieve simultaneous PA and US imaging, we implemented a controlling timing sequence using a FPGA card (myRIO-1900, NI instrument, Austin, TX, USA), as shown in Fig. 1e. In each cycle, four triggers were fired to acquire one time-resolved PA and US A-line at each point. For each cycle, the laser firing and PA signal acquisition were followed by US transmission and receiving. Two independent DAQ triggers for PA and US signal acquisition were added to improve the timing flexibility and to reduce the raw data size by avoiding acquiring unnecessary data. The A-lines were acquired at 1000 Hz, and the sampling frequency for each A-line was 250 MHz. Two-dimensional raster scanning was performed with a step size of 5 μm along the x-axis and 10 μm along the y-axis. The raw RF data was processed in MATLAB.

## Results

**Fig. 2:**
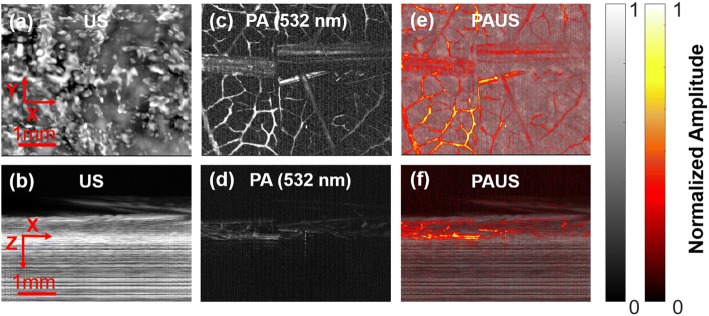
Concurrent PAUS results of a leaf phantom. The field of view (FOV) in the XY plane is 5 × 4 mm^2^. Inside the FOV, the left part is a red leaf and the right part is a green leaf. Both top view (the XY projection) and side view (the XZ projection) are provided for US (**a**, **b**), PA (**c**, **d**), and fused PAUS images (**e**, **f**)

Phantom experiment was first performed on a dry leaf with half of it dyed red and half of it dyed green. The red part of the phantom was expected to have a higher absorption with 532 nm excitation and generate a stronger PA signal. Fig. 2 shows the XY (the top row) and XZ (the bottom row) projection of both US (Fig. 2a, b) and PA images (Fig. 2c, d) at 532 nm. In contrast to US, which has low resolution in both axial and lateral axes, PA has a higher resolution of 7 μm as determined by laser beam width. However, the PA imaging penetration depth (0.6 mm) was limited by the strong scattering of the excitation photons and was thus shallower than the US penetration depth (1.6 mm). The fused PAUS image is shown in Fig. 2e, f, with PA shown in color and US in gray. By correctly setting up the time delay between laser, US, and DAQ, all US and PA images were automatically co-registered. This phantom experiment demonstrates that the concurrent PAUS imaging can simultaneously provide high resolution optical absorption information from the PA imaging and deep acoustic scattering information from the US image.

**Fig. 3:**
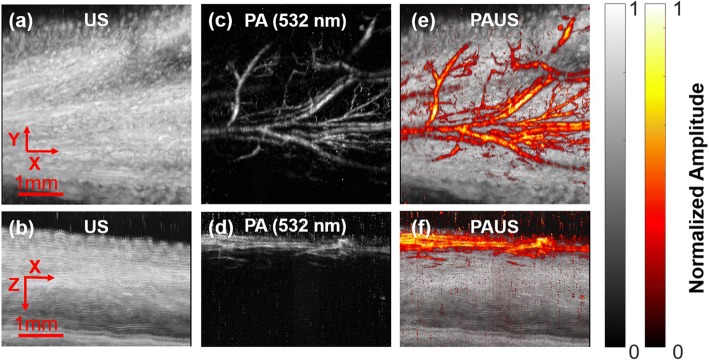
Concurrent PAUS of a mouse hind paw. In vivo PAUS results of a mouse’s hind paw, showing the XY and XZ projections for US (**a**, **b**), PA (**c**, **d**), and fused PAUS (**e**, **f**). The FOV in the XY plane is 4.8 × 4 mm^2^

To further validate this PAUS system, the hind paw of a mouse was imaged in vivo. The mouse was anesthetized with 1.5% (*v*/v) isoflurane and placed under a small water tank with a transparent bottom for acoustic coupling. The mouse’s hind paw was covered by a thin layer of ultrasound gel and fixed on the sample holder. Fig. 3 shows the XY and XZ projections for both US (Fig. 3a, b) and PA images at 532 nm (Fig. 3c, d). Endogenous hemoglobin was the primary contrast agent for the PA microvasculature imaging. In Fig. 3, blood vessels near the skin surface were clearly displayed on top of structural information revealed by US with 2.5 mm penetration. This animal study has clearly demonstrated the dramatically different but complementary contrast mechanisms of PA and US imaging of biological tissues.

**Fig. 4:**
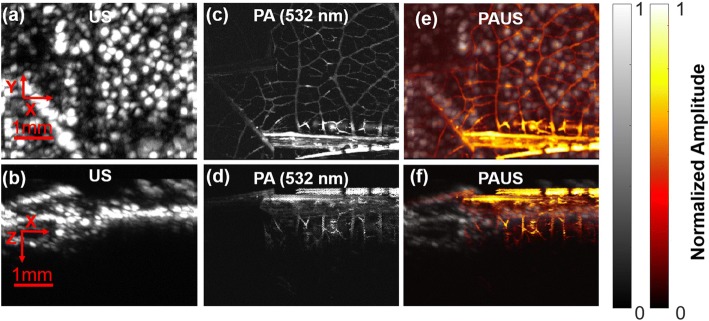
Dual-transducer PAUS of a leaf phantom. Phantom leaf results obtained by an alternative PAUS system with two single-element focused ultrasonic transducers, showing the XY and XZ projections for US (**a**, **b**), PA (**c**, **d**), and fused PAUS (**e**, **f**). The FOV for the XY plane is 5 × 4 mm^2^, and the left part is the green leaf and the right part is the red leaf

**Fig. 5:**
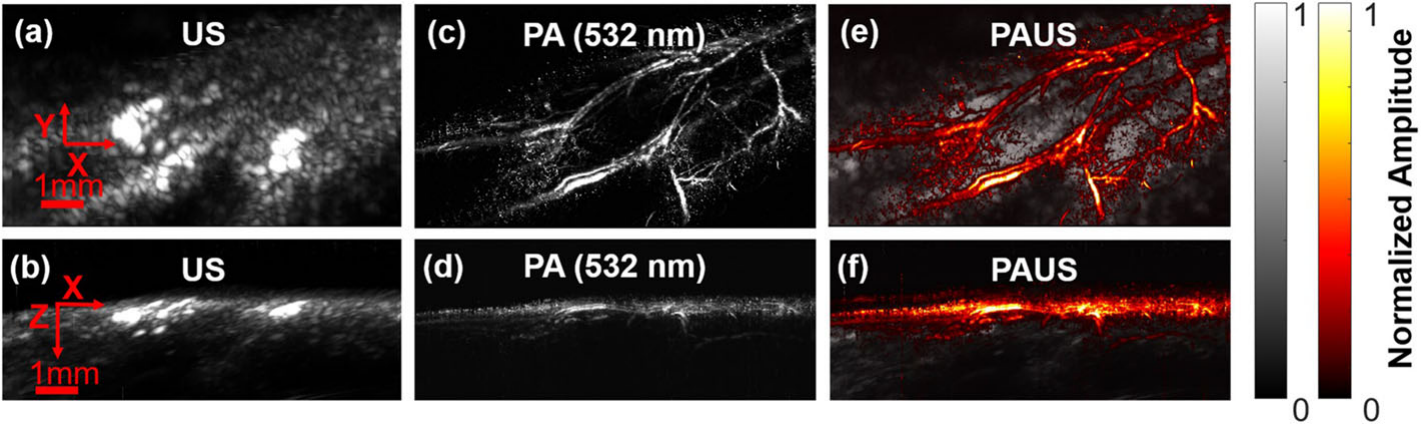
Dual-transducer PAUS of mouse hind paw. In vivo PAUS results with two single-element focused ultrasonic transducers, showing the XY and XZ projections for US (**a**, **b**), PA (**c**, **d**), and fused PAUS (**e**, **f**). The FOV in the XY plane was 9 × 5 mm^2^

In addition to the reported concurrent PAUS system using the DE-UST, which is customized and not commercially available, we also implemented an alternative system by replacing the DE-UST with two identical single-elemental focused ultrasonic transducers (V324, Olympus, Inc.) with a central frequency of 25 MHz. The two transducers were both placed pointing downward at a 45° angle with respect to the optical axis. The two acoustic foci were confocally aligned with the optical focus but off-axis. Using this alternative system, the phantom and in vivo results are shown in Fig. 4 and 5 and are similar to those obtained by the system with the DE-UST. However, different from the co-axial and reflection configuration with the DE-UST, the off-axis setup for the transducers has reduced the depth of focus of the acoustic detection and restricted the penetration depth for both PA and US. Therefore, to obtain images comparable to those obtained using the DE-UST system, z-axis scanning is necessary, which increases imaging time.

## Discussion and Conclusion

Integrated PA and US imaging has been demonstrated for needle guidance, identification of lymph nodes [11], and other in vivo structural imaging applications [26, 27]. However, the majority of existing integrated PAUS systems do not have a good enough resolution to qualify as microscopes. Here we have demonstrated a concurrent PAUS microscope that reveals optical and mechanical properties of the tissue simultaneously. Our PAUS system combines optical-resolution photoacoustic microscopy (OR-PAM) with US pulse-echo imaging, providing a high-resolution PA image that can reveal functional and anatomical information and a co-registered US pulse-echo image that can reveal general structural information [3, 28]. We have also implemented an alternative design with commercial focused ultrasonic transducer, for readers without access to the customized DE-UST. While hemoglobin was used as the endogenous chromophore for PA imaging in this study [23], multiple wavelengths could be incorporated into the system in the future for measuring oxygen saturation of hemoglobin (sO_2_) [29–31] and oxygen partial pressure (pO_2_) [24, 32, 33], as well as for molecular imaging of exogenous probes [2, 34, 35]. Our PAUS system can also be used to image microbubbles to provide blood flow velocity [36–38] and nonlinear mechanical properties [39–41]. We expect PAUS imaging to find a broad range of biomedical applications.

Nevertheless, our PAUS system still faces certain challenges. The depth of laser penetration in PA imaging is limited by the strong scattering of light in tissue. Though targeted nanoparticle contrast agents can be used to enhance the signal-to-noise ratio at greater depths [7, 42], the delivery efficiency of the targeted nanoparticles has to be further improved [43]. In addition, incorporating PA imaging into a commercial US system may increase the cost of the overall system due to the typical requirement of high-energy laser excitation [8]. Low-cost laser-diodes have been used for PA imaging [44–46], which might be a promising solution for low-cost concurrent PAUS systems.
